# Dependency of active pressure and equation of state on stiffness of wall

**DOI:** 10.1038/s41598-021-01605-8

**Published:** 2021-11-12

**Authors:** Emad Pirhadi, Xiang Cheng, Xin Yong

**Affiliations:** 1 Department of Mechanical Engineering, Binghamton University, Binghamton, NY 13902 USA; 2 Department of Chemical Engineering and Materials Science, University of Minnesota, Minneapolis, MN 55455 USA

**Keywords:** Soft materials, Theory and computation

## Abstract

Autonomous motion and motility are hallmarks of active matter. Active agents, such as biological cells and synthetic colloidal particles, consume internal energy or extract energy from the environment to generate self-propulsion and locomotion. These systems are persistently out of equilibrium due to continuous energy consumption. It is known that pressure is not always a state function for generic active matter. Torque interaction between active constituents and confinement renders the pressure of the system a boundary-dependent property. The mechanical pressure of anisotropic active particles depends on their microscopic interactions with a solid wall. Using self-propelled dumbbells confined by solid walls as a model system, we perform numerical simulations to explore how variations in the wall stiffness influence the mechanical pressure of dry active matter. In contrast to previous findings, we find that mechanical pressure can be independent of the interaction of anisotropic active particles with walls, even in the presence of intrinsic torque interaction. Particularly, the dependency of pressure on the wall stiffness vanishes when the stiffness is above a critical level. In such a limit, the dynamics of dumbbells near the walls are randomized due to the large torque experienced by the dumbbells, leading to the recovery of pressure as a state variable of density.

## Introduction

Active matter broadly refers to nonequilibrium biological or synthetic systems of motile constituents, which constantly convert chemical energy from internal or surroundings to kinetic energy and exhibit persistent directional motion^1–3^. The interaction between self-propelled active particles and system boundaries leads to an important system property termed as active pressure^4,5^, i.e., the counterpart to mechanical pressure in equilibrium systems. Active pressure can be a key driving force for membrane deformation^6–10^, which promotes host cell invasion by pathogenic bacteria into the host. It may also contribute to the expansion and migration of bacterial colonies and growing tissues^11–14^ for nutrients^15,16^. In essence, the survival of numerous active living systems relies on active pressure. Moreover, active pressure underpins some remarkable applications of active matter^17^, including microscopic ratchet motors^18^ and gears^19^.

Despite its importance, our understanding of active pressure and its relation to nonequilibrium thermodynamics remains incomplete. By controlling particle distribution throughout the system, activity influences the local density near the wall and ultimately the mechanical pressure^20^. Using the effective potential approximation, it has been shown that active pressure calculated from mechanical, virial, and thermodynamic routes do not necessarily match^21^. Thus, in contrast to passive systems in thermodynamic equilibrium, pressure is not well defined due to the lack of detailed balance in active matter^22,23^. The pioneering theoretical work of Takatori, Yan, and Brady investigated the pressure of self-propelled spherical particles and obtained an equation of state (EOS) for this active system^4,23^, which has then been confirmed by various studies^24–28^. Nevertheless, Solon et al*.* showed that the EOS is only valid for specific systems of spherical particles without torque interactions with confinement or boundaries^22^. The existence of an EOS for active particles has also been questioned in Ref.^29^. These results indicated that active pressure is controlled by boundary effects, and information about the microscopic interaction between particles and the confining walls is crucial. Fily et al*.* further demonstrated that the mechanical pressure on a wall is a function of the wall stiffness for anisotropic particles that experience torque from the wall^30^.

Experimentally, recent studies of active pressure using acoustic trap and membrane barometer showed that active pressure sensitively depends on the acoustic trap stiffness and the effective elasticity of membrane^31,32^. The effective interactions between passive particles in an active bath measured by optic tweezers also exhibited a dependence on the trap stiffness^33^. These results provide evidence that pressure of active anisotropic particles is a function of wall stiffness in real physical systems. Yet, the microscopic origin of this dependency is still poorly explored. It is also an open question whether the stiffness dependence still applies in the limit of high wall stiffness. As mechanical pressure results from the collision of particles with a wall, a fundamental understanding of how wall stiffness affects the near-wall dynamics of active particles and modulates the transferred linear momentum is critical for elucidating the origin of active pressure^34–36^.

This work explores the impact of stiffness variation on the mechanical pressure of a dry, underdamped system of self-propelled dumbbells, which possess intrinsic torque interaction with walls. We find that although pressure depends on wall stiffness for soft boundaries, this dependency vanishes as stiffness reaches high values. Through a systematic variation of the particle number density and the wall stiffness, we demonstrate that pressure follows the prediction of an EOS at high stiffnesses, even for anisotropic particles. The microscopic origin of the recovery of the EOS is further explored based on single collision events, which reveal the profound effect of single particle dynamics on the momentum transfer and the particle density near the wall. Finally, we develop a simple model to analyze an anomalous reentrant collision behavior observed for highly stiff walls and discuss the correlation between the reentrant interaction of an individual dumbbell and the non-monotonic variation of pressure with the wall stiffness. As such, our results shed light onto the unusual features of active pressure and pave the way for manipulating the pressure of active systems in various engineering applications.

## Methods

**Figure 1:**
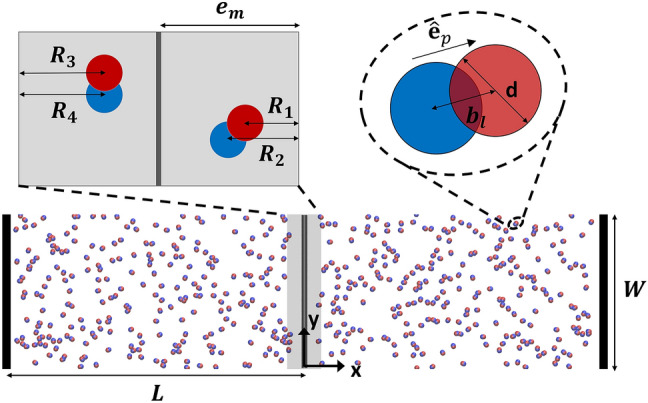
Schematic of simulation box confined by two fixed walls in the *x* direction with a mobile wall separating dumbbells into two compartments. Dumbbell geometry is depicted in the right inset. The left inset shows a schematic diagram of the mobile wall interaction zone and the instantaneous penetration depth of dumbbell beads.

We model a two-dimensional dry active system of self-propelled dumbbells to probe their active pressure. The dumbbell geometry is selected to introduce shape anisotropy typically possessed by biological microswimmers and synthetic active particles^37–40^. Inspired by previous studies^22,36^, a rectangular simulation box is separated by a mobile wall into two compartments in the *x* direction with an equal number of dumbbells in each compartment (see Fig. 1). Each dumbbell is modeled as a rigid body composed of two point particles (referred to as beads below) of mass ${m}_{b}$ constrained at a distance of ${b}_{l}$, as shown in Fig. 1. The constituent beads *i* and *j* of different dumbbells at ${\mathbf{r}}_{i}=\left({x}_{i},{y}_{i}\right)$ and ${\mathbf{r}}_{j}=\left({x}_{j},{y}_{j}\right)$ interact with each other through a pairwise Weeks-Chandler-Anderson (WCA) potential, ${U}_{ij}^{EV}=4\varepsilon \left[{\left(\sigma /{r}_{ij}\right)}^{12}-{\left(\sigma /{r}_{ij}\right)}^{6}\right]+\varepsilon$ for ${r}_{ij}=\left|{\mathbf{r}}_{i}-{\mathbf{r}}_{j}\right|<{r}_{c}$, where the cutoff radius is ${r}_{c}{=2}^{1/6}\sigma$. This repulsive potential imposes excluded volume interactions between dumbbell beads with $\varepsilon$ and ${r}_{c}$ quantifying the interaction strength and the effective diameter of the bead, respectively. Thus, the values of ${b}_{l}$ and ${r}_{c}$ determine the effective aspect ratio of the dumbbell. Although a physical swimmer is force-free and torque-free, activity of dumbbell is imparted by applying a propulsion force^41^ to each individual bead ${\mathbf{F}}_{i}^{p}={f}_{p}{\widehat{\mathbf{e}}}_{p}$. ${f}_{p}$ is a propulsion constant and the direction ${\widehat{\mathbf{e}}}_{p}=\left({\mathbf{r}}_{h}-{\mathbf{r}}_{t}\right)/\left|\left({\mathbf{r}}_{h}-{\mathbf{r}}_{t}\right)\right|$ pointing from the tail bead to the head bead, where ${\mathbf{r}}_{h}$ and ${\mathbf{r}}_{t}$ are the respective positions of the head and tail beads. The effective swimming velocity of the isolated dumbbell can thus be calculated through the overdamped Langevin equation in the absence of interparticle interactions, given by ${\mathbf{v}}_{0}={v}_{0}{\widehat{\mathbf{e}}}_{p}$. The swimming speed is ${v}_{0}={f}_{p}/{\gamma }_{b}$ with ${\gamma }_{b}$ being the friction coefficient with the background medium (e.g., substrate).

The mobile wall spans the entire *y* dimension of the box and has a mass of ${m}_{w}$. It applies a soft-core repulsion to a nearby dumbbell bead *i*, expressed as ${U}_{i}^{MW}={k}_{L, R}{({r}_{p}-{e}_{m})}^{2}$. Here, ${r}_{p}=\left|{x}_{i}-{x}_{w}\right|$ is the normal distance between the bead and the wall with ${x}_{w}$ being the instantaneous wall position. ${e}_{m}$ represents the range of repulsion and can be considered as the effective thickness of the wall^36^. The strengths of repulsion between the mobile wall and the dumbbells in the left and right compartments can be independently controlled by ${k}_{L}$ and ${k}_{R}$, respectively. The repulsion parameters thus quantify the stiffness of the wall. The motion of the mobile wall is driven by the interactions with dumbbells on both sides of the wall but is also subjected to the background friction. The simulation box is confined in the *x* direction by two additional fixed walls. Similar to the mobile wall, a fixed wall located at the edge of the simulation box ${x}_{FW}$ exerts a harmonic repulsion ${U}_{i}^{FW}={k}_{FW}{({r}_{p}-{e}_{f})}^{2}$ to a dumbbell bead *i* when $\left|{x}_{i}-{x}_{FW}\right|<{e}_{f}$. The repulsion parameter ${k}_{FW}$ is tuned to prevent dumbbells from leaving the simulation box. The periodic boundary condition (PBC) is applied in the *y* direction.

The dynamics of the system is governed by modified Langevin equations applied to the mobile wall and each dumbbell beads,

1 $${m}_{w}{\ddot{x}}_{w}=-{\gamma }_{w}{\dot{x}}_{w}+\sum_{i}{F}_{i}^{W}$$

and

2 $${m}_{b}{\ddot{\mathbf{r}}}_{i}=-{\gamma }_{b}{\dot{\mathbf{r}}}_{i}-{\nabla }_{i}{U}_{i}+{\mathbf{F}}_{i}^{p}$$

where ${F}_{i}^{W}={-\nabla }_{x}{U}_{i}^{MW}$, and ${U}_{i}=\sum_{j}{U}_{ij}^{EV}+{U}_{i}^{MW}+{U}_{i}^{FW}$. The summation in Eq. (1) runs for all beads *i* within the cutoff distances from the mobile and fixed walls, while the summation in Eq. (2) runs for all neighbor beads *j* within the cutoff of the WCA potential from bead *i*. The random noise is not considered in this work due to its negligible impact on the effective diffusion of interacting active particles^42,43^. The equations of motion are solved using the velocity-Verlet algorithm. The simulations are implemented using the particle simulation code LAMMPS (Large-scale Atomic/Molecular Massively Parallel Simulator)^44^ with in-house modifications.

In this work, we set the cutoff radius and potential well depth of the WCA potential as the characteristic length and energy scales, respectively. The characteristic mass is the mass of a dumbbell ${m}_{d}=2{m}_{b}$. The characteristic time scale ${t}_{c}$ can then be defined as ${t}_{c}=\sqrt{{m}_{d}{{r}_{c}}^{2}/\varepsilon }$. For simplicity, ${m}_{d}$, ${r}_{c}$, $\varepsilon$, and ${t}_{c}$ are all set to one, and the simulation parameters are presented in reduced units. The dimension of the simulation box is set to $2L\times W$, with the mobile wall initially located at ${x}_{w}=0$. To obtain statistically reliable data^36^, the number of dumbbells in each compartment is set to ${n}_{d}=250$ unless stated otherwise. A higher number of dumbbells would increase simulation cost without providing additional insight into the problem. We set $W=70$ to be about two orders of magnitude larger than the dumbbell length to minimize the finite-size effects arising from possible PBC artifacts. The lateral dimension of the initial compartment is set to $L=148$ to obtain a norminal packing fraction of 10%. The packing fraction of the system is defined as the fraction of the simulation box that is occupied by dumbbells, $\phi =n\left(2{A}_{b}-{A}_{int}\right)/\left(2LW\right)$, where ${A}_{b}$ is the area of each bead and ${A}_{int}$ is the overlapping area of two beads of a dumbbell^45^. Higher packing fractions could result in wall induced aggregation^24^ as well as motility-induced phase separation^46^, which would drastically change the nature of the system. Although a lower packing fraction results in similar behavior, it would increase the fluctuation of the system and require longer and more expensive sampling. To allow comparison with previous studies^36^, the mass of the mobile wall is set to ${m}_{w}=2.0$, and the friction coefficients of the bead and the wall are set to be ${\gamma }_{b}=0.5$ and ${\gamma }_{w}=2.0$, respectively. The bond length of the dumbbell is ${b}_{l}=0.5$. Unless stated otherwise, the swimming speed is set to be ${v}_{0}=2$. The mobile wall thickness is set to ${e}_{m}=8$ the same as Ref.^36^. ${k}_{L}$ and ${k}_{R}$ vary in the range of 0.2 to 300. The fixed walls have an interaction range of ${e}_{f}=5$ and a repulsion strength of ${k}_{FW}=50$. To accurately resolve the detailed dynamics of the system, we choose a very small timestep in this study $\Delta t=5\times {10}^{-5}$. Additional simulations with smaller timesteps were performed to ensure consistent behaviors. The total time of a typical simulation of the bulk system is $t=5000$.

## Results

### Dynamic behavior of the bulk system of active dumbbells

**Figure 2:**
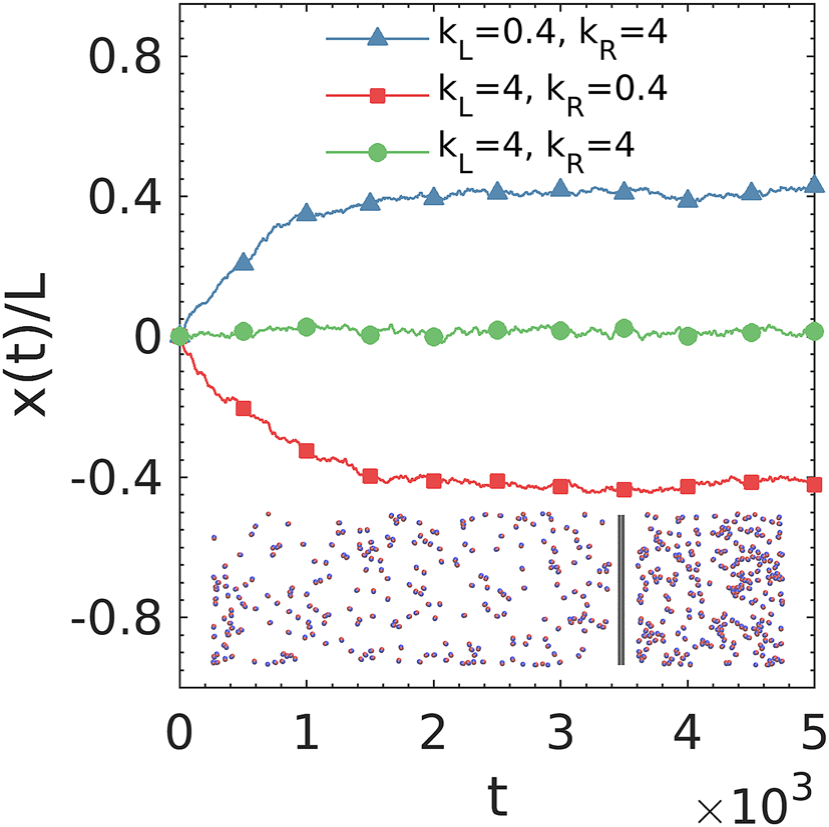
Time evolution of normalized position of the mobile wall in the simulations with three different combinations of wall stiffnesses. Inset is a representative snapshot at the end of simulation for the case of ${k}_{L}=0.4$ and ${k}_{R}=4$. The profiles are averaged across five independent runs with different initial distributions of dumbbells.

We first explore the dynamics of a bulk system of active dumbbells. We conduct a mobile-wall experiment^22^ to study the effect of wall interactions on the active pressure in our system. Two sides of the mobile wall have different stiffness parameters and hence exert asymmetric repulsion to the dumbbells in the different compartments. We consider the mechanical pressure of active dumbbells as the summation of forces that dumbbells apply to the wall divided by the wall area. The movement of the mobile wall thus reflects the relative difference of mechanical pressure in the two compartments. Figure 2 shows that the mobile wall moves toward the side experiencing stiffer wall interaction, which indicates that increasing wall stiffness reduces the mechanical pressure of self-propelled dumbbells. This behavior is consistent with previous studies and demonstrates that the pressure of active systems is influenced by their interactions with the confining boundaries^22,30,36^.

**Figure 3:**
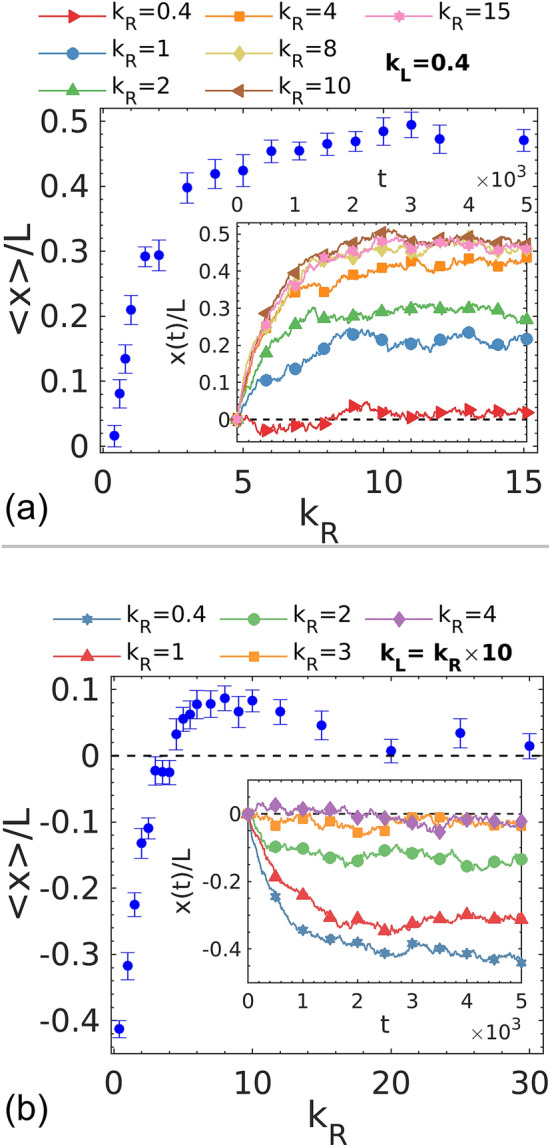
Ensemble average of normalized wall position as a function of ${k}_{R}$ for the simulations with (**a**) ${k}_{L}$ remaining constant at 0.4 and (**b**) the ratio of stiffness ${k}_{L}/{k}_{R}$ remaining constant at 10. Average wall position (represented by the angular bracket) is sampled during the equilibrium stage, defined as the last 2500 time units of a simulation. The instantaneous wall positions for different ${k}_{R}$ are plotted in the inset. The position corresponding to the center of the box in the *x* direction is marked with a dashed line in (**b**). Five independent runs with different initial configurations are performed for the analysis. Error bars represent the standard deviation.

To better understand the relationship between pressure and wall interaction, we perform simulations with systematic variations in the wall stiffness. We first increase the right-side stiffness, ${k}_{R}$, gradually from 0.4 to 15 while keeping the left-side potential, ${k}_{L}$, constant at 0.4. It is expected that the equilibrium position of the mobile wall would progressively move to the right as ${k}_{R}$ increases, which represents a greater imbalance in pressure between the two sides. As illustrated in Fig. 3a, the average value of normalized wall position first increases as ${k}_{R}$ increases, corresponding to the movement of the wall to the right and the shrinking of the right compartment. However, a plateau can be clearly observed when ${k}_{R}>5$. Further increase in ${k}_{R}$ results in only fluctuations of the equilibrium wall position between 0.45 and 0.5. This indicates that the magnitude of mechanical pressure on the right side saturates to a low plateau around ${k}_{R}=6$. However, it would be premature to conclude that pressure becomes independent of wall stiffness when increasing beyond a threshold based on only this test. Namely, when the wall moves toward the right, the dumbbell density in the right compartment correspondingly increases. When density is high enough, even a small rise in density could cause a huge elevation in pressure. The density-induced increase could compensate for the pressure decrease originated from the change in the wall interaction and prevents the wall from moving further toward the right.

Therefore, to verify the behavior we observed, we design a second test in which the ratio of stiffness ${k}_{L}/{k}_{R}$ is kept constant at 10 while the absolute stiffness is increased accordingly on both sides. Interestingly, in the simulations with the right wall potential higher than 4, the mobile wall stays roughly at the middle of the simulation box, which means that the mechanical pressures on the left and right are balanced (Fig. 3b). Notably, the dumbbell densities are approximately equal on both sides of the wall, and any density effect on pressure should vanish in these cases. Therefore, the only factor that could influence the mechanical pressure is the interaction between the wall and dumbbells. The results of these two tests demonstrate that the dependency of the mechanical pressure on the wall-dumbbell interaction indeed vanishes at high wall stiffnesses. The movement of the wall implies the qualitative behavior of the mechanical pressure of active dumbbells. Starting from a system with an extremely soft wall (i.e., ${k}_{R}=0.4$), the pressure reduces with increasing wall stiffness until it converges to a minimal amount. After reaching the minimum, any increase in wall stiffness does not change pressure anymore. In such a limit, the mechanical pressure becomes independent of the interaction of active particles with the boundaries, which is an indication that an EOS may exist.

**Figure 4:**
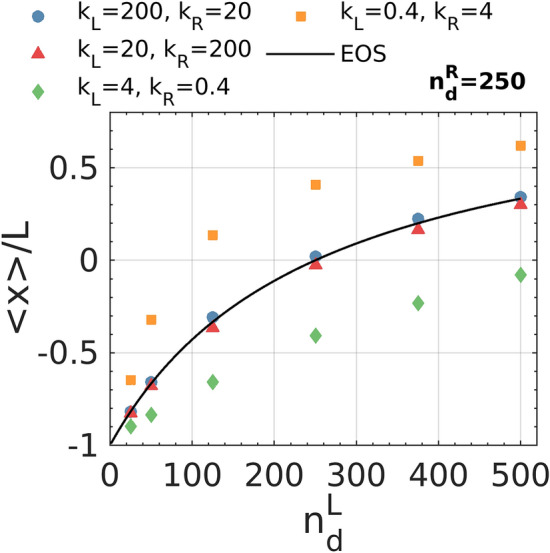
Ensemble average of normalized wall position as a function of the number of dumbbells ${n}_{d}^{L}$ in the left compartment while ${n}_{d}^{R}$ is kept constant. The prediction of a pressure equation of state for the equilibrium position of the wall is plotted in a black line.

We employ an additional mobile wall experiment to explore the recovery of active pressure as a state variable by distinguishing the dependency of pressure on density and stiffness. In this set of simulations, we set the number of dumbbells in the right compartment to be constant at ${n}_{d}^{R}=250$, and systematically varies the number of dumbbells in the left compartment ${n}_{d}^{L}$ from $25$ to $500$ for different sets of wall stiffnesses. Swim velocity and friction with the background are equal between the two compartments. Thus, the mobile wall is expected to move until the number densities of two sides become equal if the pressure of active dumbbells is a state function of density and independent of wall interaction. The equilibrium wall position for any stiffness asymmetry can thus be calculated by simply matching the number densities of the two compartments, representing the result of the existence of a pressure EOS. Figure 4 shows that for highly stiff walls, the average position of the wall agrees with the prediction of the EOS, and the ensemble averaged position of the wall does not change by swapping the stiffness asymmetry. This result confirms that pressure does not depend on microscopic interactions of particles with the wall in a limit of high wall stiffness. Notably, for softer walls, the average wall position and thus pressure depends on both dumbbell density and wall stiffness.

### The relation between active pressure and near-wall microstructure

We utilize a method that we term fictitious wall method to quantify pressure. Here, we consider a physical wall with symmetric repulsion fixed in the simulation box. We introduce an imaginary wall at a specific location of the box to calculate the local pressure. All dumbbells within the cutoff range of this imaginary wall would apply forces to the wall if it were a real wall. The summation of the virtual forces from the interacting dumbbells divided by the area of the wall defines an instantaneous pressure (see Supplementary Information for more details). Essentially, this value represents a mechanical pressure that a real wall would experience at the moment of introduction to the system before the wall induces any changes in the dumbbell distribution. The calculation shows that near-wall pressure decreases with an increase in stiffness in lower limits of ${k}_{w}$. However, the change in near-wall pressure becomes less discernible as ${k}_{w}$ increases and a convergent trend to a critical value is appreciable (see Fig. S1). This result provides further evidence that except for cases with a very soft wall, the mechanical pressure of active dumbbells is independent of the stiffness, which agrees with the recovery of an EOS observed in stiff limits.

**Figure 5:**
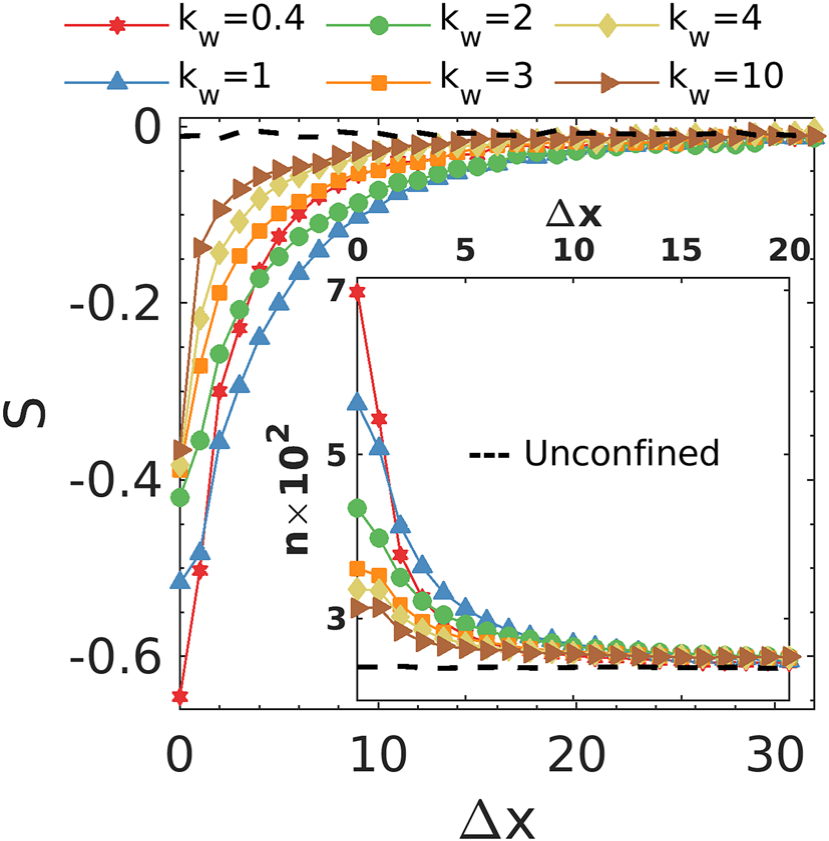
Orientational order parameter of dumbbells as a function of distance from the wall. The number density of dumbbells as a function of distance from the wall is plotted in the inset. For comparison, the dashed lines present the results of an unconfined system with full PBCs in both *x* and *y* directions. The swimming velocity is set to be ${v}_{0}=1$ for these simulations.

Mechanical pressure is directly related to the number of dumbbells interacting with the wall^20^. The number density profile in Fig. 5 shows that the near-wall density is higher for the softer walls. Consequently, the softer walls experience higher pressure from dumbbells compared to the stiffer walls. To answer how ${k}_{w}$ influences the near-wall density, we introduce the 2D nematic order parameter $S=2<{\mathrm{cos}}^{2}\theta >-1$ to quantify the dumbbell orientation. Here $\theta$ is the instantaneous angle between the dumbbell axis and the normal to the wall, and the angular bracket represents the ensemble average. The value of *S* ranges between -1 to 1 and characterizes the average orientation of dumbbells. In particular, $S=-1$ corresponds to the case where dumbbells align parallel to the wall, while the value of 1 indicates that dumbbells are perpendicular to the wall. A system of dumbbells without any preferential orientations will result in $S=0$. Figure 5 shows that *S* increases from a negative value greater than -1 to 0 as the distance from the wall increases. This indicates the development of alignment near the wall due to the repulsion from the wall. The comparison reveals that the value of *S* near the wall increases as ${k}_{w}$ increases, which corresponds to the disruption of alignment. In other words, a weaker wall repulsion promotes the alignment of dumbbells with the wall. A higher degree of alignment results in lower swim velocity in $x$ direction opposite to the wall. Consequently, the particle flux toward the wall would be higher than the outgoing flux, and the particle density and pressure would be higher near the wall. Interestingly, *S* near the wall appears to converge to a critical value with the increase of ${k}_{w}$. This behavior could be associated with the converging pressure observed in Fig. S1. Note that the mechanical pressure on the wall is also proportional to the total linear momentum that each dumbbell transfers to the wall. Below, we demonstrate that the torque induced alignment of dumbbells with the wall enhances the linear momentum transfer.

### Single dumbbell interaction

To elucidate fundamental mechanisms of the intriguing behaviors observed in the bulk system, we model the interaction of a single dumbbell with the wall and probe the detailed dynamics of collision events, which contribute collectively to the pressure. This simple system allows us to remove the complexity arising from the dumbbell-dumbbell interaction and isolate the dumbbell-wall interaction for detailed analyses. Herein, the wall position is fixed at the edge ($x=L$) of the simulation box with the PBC applied in the *y* direction as in the bulk system. The self-propelled dumbbell is initially placed outside the cutoff distance of wall repulsion and starts moving toward the wall. The simulation continues until the dumbbell completes its interaction and moves away from the wall indefinitely. The wall has the same thickness ${e}_{m}$ and stiffness ${k}_{w}$ as the mobile wall in the bulk system.

We find that the collision dynamics depend strongly upon the entrance angle ${\theta }_{i}$, which is defined as the angle between the dumbbell axis and the normal to the wall (pointing inward) when the dumbbell enters the wall interaction zone. For each ${k}_{w}$, we systematically explore the behavior of dumbbells by varying ${\theta }_{i}$ in the range of $5^\circ\le {\theta }_{i}\le 85^\circ$ with a 5° increment. The mechanical pressure is directly related to the total amount of linear momentum $\Delta p$ that dumbbells transfer to the wall. $\Delta p$ for each collision is calculated by $\Delta p= \overline{F }\times \tau$, where $\tau$ is the duration of interaction and $\overline{F }$ is the average force in the *x* direction during the interaction.

**Figure 6:**
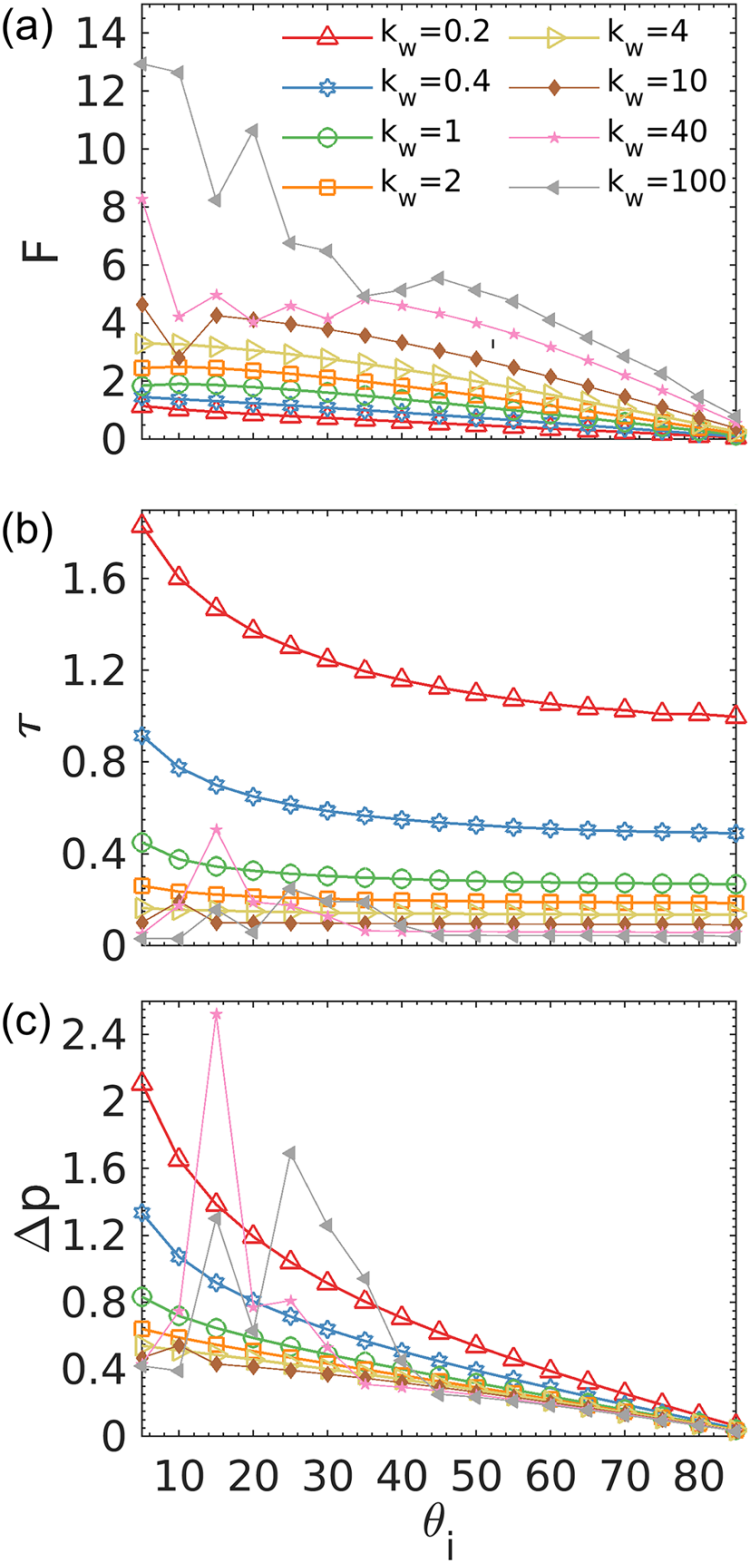
(**a**) Average force, (**b**) duration of interaction, and (**c**) total transferred linear momentum as functions of the entrance angle for a single dumbbell interaction.

It is intuitive that stiffer walls apply larger forces to dumbbells, which is confirmed by our data (Fig. 6a). However, stiffer walls result in shorter interaction durations, as shown in Fig. 6b. Therefore, there exists a competition between the variations in $\overline{F }$ and $\tau$, which determines the changes in $\Delta p$ and accordingly pressure. Figure 6c indicates that the effect of ${k}_{w}$ on $\tau$ overpowers that of $\overline{F }$, which results in stiffer walls exhibiting lower momentum transfer and pressure. This dominance gradually vanishes as ${k}_{w}$ further increases. Consequently, the total amount of transferred momentum $\Delta p$ to the wall converges to a critical value with increasing stiffness, which agrees with the behavior of mechanical pressure we observed in the bulk system. This quantification of dumbbell-wall collision supports our finding that the wall effect on mechanical pressure of active dumbbells diminishes as the stiffness of the wall increases.

To understand why the duration of interaction is longer for softer walls, we scrutinize critical dynamic quantities at play in the dumbbell-wall interaction. Fig. S2 plots the evolution of these quantities for a single collision event at a moderate entrance angle. For a particle without activity, the normal force from the wall is the only factor that determines the duration of the interaction. In contrast, for active particles, a change in swimming direction is required to leave the wall. The reorientation time scale is the key factor behind the swimming direction change of a self-propelled spherical particle. For a smooth-swimming dumbbell without Brownian noise, the important parameter is the instantaneous torque that the wall applies to the dumbbell, $T$, which governs the rotation of the dumbbell and dictates $\tau$. Torque interactions from softer walls result in lower angular velocity $\omega$ of a rotating dumbbell, allowing it to stay in the interaction zone for longer times. This also explains why in the bulk system, dumbbells are more aligned with softer walls with higher near-wall number densities. Here, the signs of *T* and $\omega$ follows the right-hand rule (i.e., positive/negative *T* leads to counter-clockwise/clockwise rotation with positive/negative $\omega$). The magnitude of $T$ is determined by the dumbbell orientation and the difference in the repulsive forces on the head and tail beads from the wall. The evolution of the former depends mainly on the entrance angle ${\theta }_{i}$ whose distribution should be similar between different cases of the bulk system. The latter, on the other hand, is a function of ${k}_{w}$ which varies from case to case. The dependency of $T$ on ${k}_{w}$ denotes that walls with higher stiffnesses apply larger $T$ to dumbbells. This makes dumbbells rotate more, interact with the wall for shorter times (lower $\tau$), transfer less linear momentum $\Delta p$, and eventually apply less mechanical pressure to the wall. As ${k}_{w}$ further increases, Fig. 6c demonstrates that the monotonic behavior breaks down at certain values of ${\theta }_{i}$. This behavior is attributed to an anomalous interaction dynamic in which the dumbbell exhibits multiple collisions with the wall in a very short time before leaving the wall indefinitely. We analyze this behavior in detail and elucidate its influence on momentum transfer and pressure generation.

### Reentrant collision event

**Figure 7:**
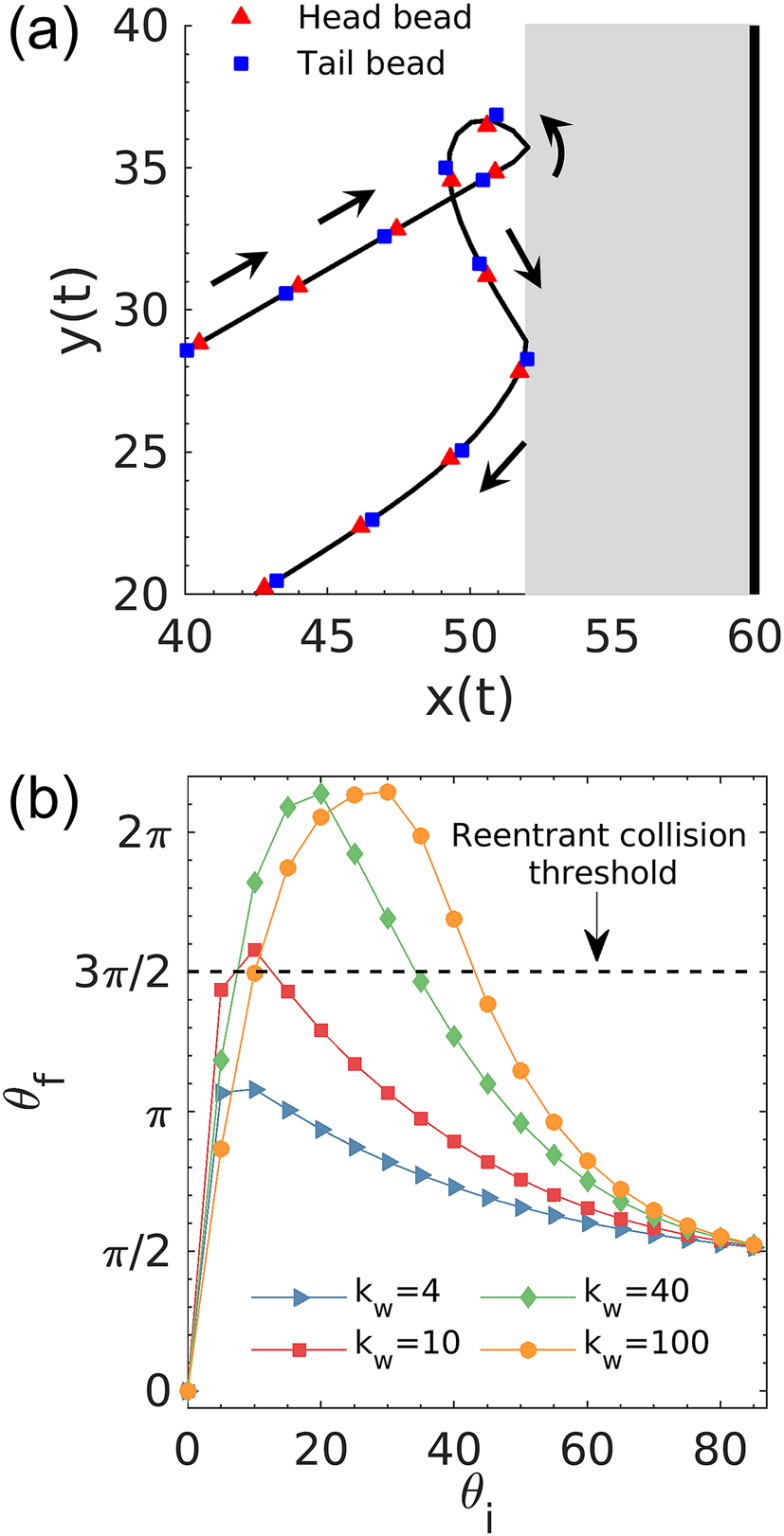
(**a**) Representative trajectory of a dumbbell with ${\theta }_{i}=30^\circ$ exhibiting the reentrant collision dynamics. The wall is positioned at $x=60$ with ${k}_{w}=40$ and ${e}_{f}=8$. (**b**) Final orientation angle of the dumbbell with respect to the wall normal after the first collision event as a function of initial entrance angle. When reentrant collision motion happens, ${\theta }_{f}$ becomes the entrance angle for the subsequent collision. The dashed line marks the threshold for the onset of reentrant collision.

The non-monotonic behaviors of average force, interaction duration, and momentum transfer observed for stiff walls (${k}_{w}\ge 10)$ in Fig. 6 is associated with the onset of anomalous collision dynamics, resembling the one observed in previous studies^36^. A representative trajectory of a dumbbell undergoing this anomalous behavior is shown in Fig. 7a. In particular, after hitting the wall, a dumbbell may rotate too much to face toward the wall again during each collision and therefore reenters the wall interaction zone and collides the wall again before it moves away from the wall indefinitely (Video S2). The reentrant collision effectively increases the duration of interaction and the rate of momentum transfer to the wall defined in a specific time span. In the bulk system, the onset of reentrant collision also results in an increase in the collision rate and the elevation of pressure. The torque from the wall $T$ induces rotation and facilitates this motion, while the background friction reduces the angular velocity and prevents it. Our data indicate that $T$ is a function of wall stiffness ${k}_{w}$, dumbbell bond length ${b}_{l}$, swimming speed ${v}_{0}$, and the entrance angle ${\theta }_{i}$ of the first interaction. These parameters govern the evolution of dumbbell orientation $\theta (t)$ by controlling the angular acceleration. Note that a considerable portion of dumbbell rotation takes place outside of the wall interaction zone. This implies that inertial effects play an important role in the reentrant collision. Therefore, this phenomenon has not been well documented in previous studies largely focused on overdamped systems.

The rotation of the dumbbell is governed by Newton’s second law for rotation $Id\omega /dt=T$, where $I$ and $\omega$ are the moment of inertia and the angular velocity of the dumbbell, respectively. Outside the wall interaction zone, only friction contributes to the torque. Given the knowledge of angular velocity ${\omega }_{e}$ when the dumbbell leaves the interaction zone and the corresponding exiting angle ${\theta }_{e}$ for the first collision event, we can predict the onset of reentrant collision based on a final angle ${\theta }_{f}$, which is defined when $\omega$ decreases to 0. Using the outlet ${\omega }_{e}$ and ${\theta }_{e}$ obtained in the simulation, ${\theta }_{f}$ can be calculated as ${\theta }_{f}={\theta }_{e}+\left(m_{b}/{\gamma }_{b}\right){\omega }_{e}$. If ${\theta }_{f}$ is greater than $3\pi /2$, the dumbbell has been rotated enough to face toward the wall and will hit it again. The results of this calculation match the simulation data of ${\theta }_{f}$. Figure 7b compares the onset of reentrant collision events between different ${k}_{w}$ for various initial entrance angle ${\theta }_{i}$ in the collision simulations. Points above the dashed line correspond to the onset of reentrant collision events. Notably, dumbbells with different intervals of ${\theta }_{i}$ exhibit reentrant motion, and some of them may even hit the wall more than twice before leaving indefinitely (Video S3). This makes the exact prediction of active pressure extremely complicated. It is also important to note that this simple prediction of reentrant collision is based on the single-dumbbell system, and the introduction of Brownian noise and interparticle interactions will alter the dumbbell dynamics significantly^47^.

## Discussion and conclusions

In this paper, we applied numerical simulation to investigate the influences of wall stiffness on the mechanical pressure generated by anisotropic self-propelled particles with intrinsic torque interaction. The torques applied to particles by the wall influence the rotational evolution of particles, which affects the distribution of particles throughout the domain. Our results show that the mechanical pressure near the wall decreases with increasing wall stiffness, and more importantly, reaches a constant plateau above a certain stiffness. This means that even for anisotropic particles like dumbbells, active pressure is not always influenced by interaction with the confining wall. Our study indicates that for a characteristic system of strongly interacting particles without random noise, there exists a regime in which active pressure is not a boundary effect.

The dynamics of a single collision event was explored in detail to uncover the microscopic origin of the relation between pressure and wall stiffness. The mechanical pressure is linked to the total linear momentum transfer between dumbbells and the wall during each interaction. We find that the amount of torque that the wall applies to dumbbells is a function of both the entrance angle and wall stiffness. Torque alters the total linear momentum by controlling rotation of the dumbbell and duration of the interaction. Our results indicate that the total linear momentum transferred to the wall during one collision converges to a minimum amount through increasing the stiffness, showing a similar trend as the mechanical pressure.

The collective impact of single particle dynamics on pressure arises from density distribution, or essentially particle accumulation near the wall. The rotational time scale near the wall controls particle escape time, which is a critical factor involved in the accumulation of anisotropic particles near the wall. Wall stiffness is correlated to the rotational time scale of dumbbells by controlling the magnitude of the torque that is applied to them. Higher torque reduces the rotational time scale, while the swimming time scale, dictated by the propulsion force, remains constant. These two time scales together determine the rotation and translation of dumbbells near the wall. Highly stiff walls induce a large torque, which makes the rotational time scale to be smaller than the swimming time scale. As a result, rotation will dominate self-propulsion, and we speculate dumbbells orientation near the wall becomes approximately random. The results of Joyeux and Bertin^36,47^ showed that the notion of mechanical pressure could be recovered as a result of the interplay between rotation and self-propulsion for a system of active dumbbells with very low background friction or strong Brownian noise. Decreasing friction or increasing noise intensity will both reduce the rotational time scale. Therefore, increasing wall stiffness yields a similar effect as varying friction or noise.

According to the kinetic theory, particle density influences pressure via controlling collision rate. Thus, single particle dynamics near the wall influences the mean collision rate through modulating the near-wall density. On the other hand, the amount of linear momentum transferred to the wall during each collision is also determined by single particle dynamics. Confining walls apply torque to anisotropic active particles, which strongly influences their dynamics. As a result, particle density, momentum transfer, and eventually pressure would vary by the wall stiffness. For highly stiff walls, both contributions of single particle dynamics to pressure converge to a critical value, likely because of randomization of particle orientation. Consequently, dependency of pressure on single particle dynamics vanishes for high limits of stiffness. As a result, pressure becomes a function of only bulk properties of the system, which indicates that an EOS could be found.

**Figure 8:**
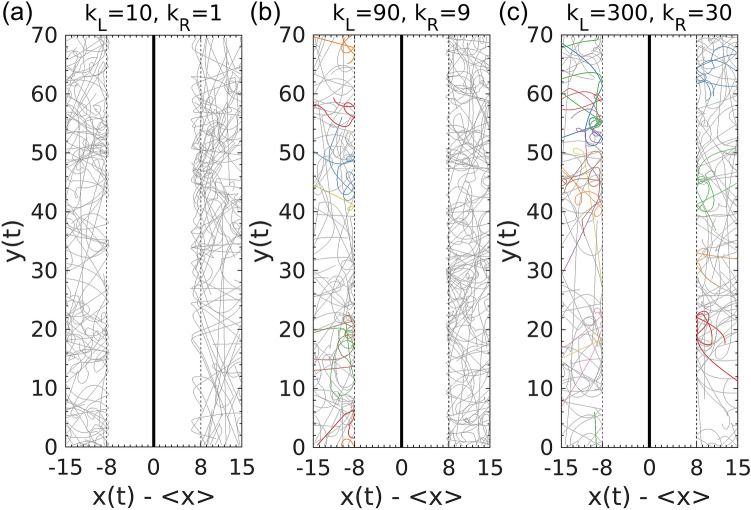
Trajectories of individual dumbbells interacting with walls with different asymmetric interactions for 40 time units intervals. (**a**–**c**) correspond to the low, intermediate, and high stiffness regimes shown in Fig. 3b. Dumbbells exhibiting reentrant collision events are colored differently. The walls are fixed at respective equilibrium positions for different wall stiffnesses.

Anomalous particle trajectories were observed for extremely stiff walls. Namely, if a dumbbell gains a large amount of angular momentum during the interaction, its rotation will result in one or more reentrant collisions with the wall. The onset of this anomaly depends on the stiffness of wall interaction and the entrance angle of the initial collision. The dumbbell trajectories in Fig. 8a confirm that reentrant collision does not occur for small ${k}_{R}$. We further elucidate how reentrant collision could affect active pressure. Figure 3b shows a non-monotonic trend, where for a small range of stiffness ($4<{k}_{R}<20$), the pressure on the stiffer side of the wall is higher. This unusual trend of pressure variation is not attributed to the stiffness-induced change in the transferred linear momentum for each collision but instead caused by the difference in the collision rate in the presence of the reentrant collisions, which are more probable to occur for the stiffer walls. As shown in Fig. 8b and Video S4, the reentrant collisions only take place or occur more frequently on the left side of the wall (the stiffer side) in the discussed interval of ${k}_{R}$. Consequently, the mechanical pressure becomes higher on the stiffer side. However, as shown in Fig. 8c, the onset of reentrant collision becomes comparable on both sides when the stiffness increases beyond this range of ${k}_{R}$. Therefore, the appearance of this anomalous motion would not affect the pressure in the stiff limit.

In summary, our understanding of active pressure remains incomplete and warrants more research, in particular experimental studies, to provide additional insight into the complex interaction between active particles and walls of different stiffnesses. Among many uncharted areas, we envision our future work to be focused on the active pressure of flexible particles and particles with a directional reversal.

## Supplementary Information

Supplementary Information 1.

Supplementary Video 1.

Supplementary Video 2.

Supplementary Video 3.

Supplementary Video 4.
