# Supplementary material for: Dependency of active pressure and equation of state on stiffness of wall
Source: Sci Rep. 2021 Nov 12;11:22204. doi: 10.1038/s41598-021-01605-8 (PMC8590019; doi:10.1038/s41598-021-01605-8)
Supplement: Supplementary file 1 — Supplementary Information 1. [file 41598_2021_1605_MOESM1_ESM.pdf]

Supplementary Information for "Dependency of Active Pressure and Equation of State on Stiffness of Wall"

Emad Pirhadi[1], Xiang Cheng[2], Xin Yong[1*]

[1] Department of Mechanical Engineering, Binghamton University, Binghamton, NY 13902, USA.

[2] Department of Chemical Engineering and Materials Science, University of Minnesota, Minneapolis, MN 55455, USA.

Fictitious wall method for quantifying active pressure

In this method, the physical wall position is fixed without any asymmetry in the stiffness ($k_L = k_R = k_w$), and we apply the PBCs in both x and y directions. To avoid the appearance of reentrant collision events of dumbbells interacting with the wall for the full range of k_w, a lower swimming speed $v_0 = 1$ is used. We discuss this anomalous collision and its influence on pressure in detail in Section "Reentrant collision event". To calculate the pressure at a specific location in the domain, we introduce an imaginary wall there. All dumbbells within the cutoff range of this wall would apply forces to the wall if it were a real wall. The summation of the virtual forces from the interacting dumbbells divided by the length of the wall defines an instantaneous pressure $P(x,t) = \frac{1}{L_y} \sum k_0 R_i(t)$ for that position. Here $R_i(t)$ is the instantaneous penetration depth of dumbbell bead i inside the wall cutoff (Figure 1). We set $k_0 = 1$ to be a constant and the fictitious wall serves as an external probe independent from the influence of real wall. The reference pressure P_0 is defined as the bulk pressure of an unconfined system with full PBCs in both x and y directions, which is calculated by averaging the local pressure in x and over time in the unconfined system. Figure S1 plots the pressure profile averaged over 19000 time units for different wall stiffness. We observe behavior consistent with previous studies that for systems with a soft wall, the mechanical pressure near the wall is larger than that far away from the wall[1,2]. Notably, the wall stiffness has a minor effect on the local pressure far away from the wall, likely due to the change of effective packing fraction as the effective excluded volume of the wall is different.

References

1. Speck, T. & Jack, R. L. Ideal bulk pressure of active Brownian particles. *Phys. Rev. E* **93**, 062605 (2016).
2. Omar, A. K., Wang, Z.-G. & Brady, J. F. Microscopic origins of the swim pressure and the anomalous surface tension of active matter. *Phys. Rev. E* **101**, 012604 (2020).

Supplementary Figures

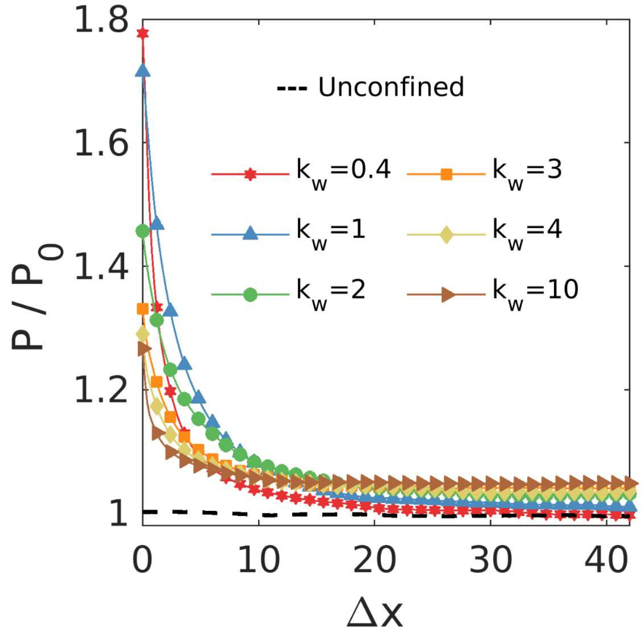

Fig. S1. Normalized local mechanical pressure as a function of distance from the wall. The dashed line represents the result of an unconfined, fully periodic system. The swimming velocity is set to be $v_0 = 1$ for these simulations.

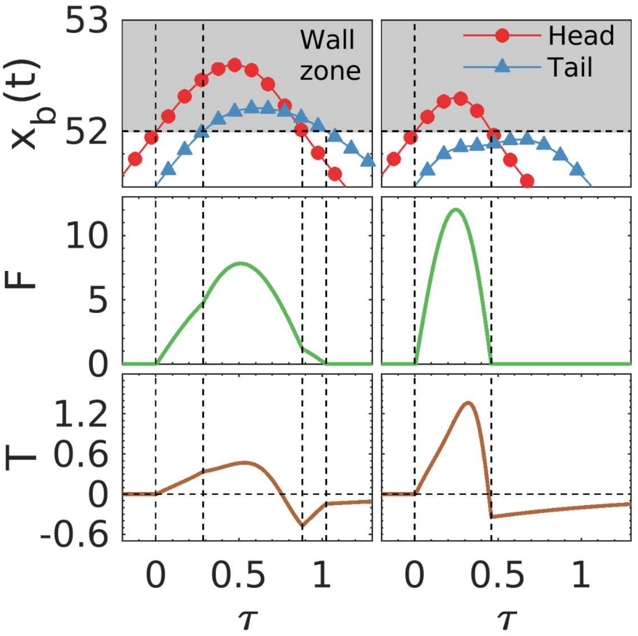

Fig. S2. Instantaneous *x* positions of dumbbell beads, total repulsive force that wall applies to the dumbbell, and total torque on the dumbbell during a single collision event with entrance angle $\theta_i = 15$. The wall stiffnesses are $k_w = 10$ and $k_w = 40$ for the left and right cases, respectively. The shaded area marks the wall interaction zone. Instants when each bead enters and leaves the wall interaction zone are marked with vertical dashed lines.

Supplementary Videos

Video S1. Simulation video of the mobile wall test with $k_L = 4$ and $k_R = 0.4$.

Video S2. Dynamics of a single dumbbell exhibiting a reentrant collision event during interaction with a wall of stiffness $k_w = 40$ at $\theta_i = 30$.

Video S3. Simulation video showing a representative reentrant collision during which the dumbbell hits the wall multiple times before moving away indefinitely. The wall stiffness is $k_w = 40$ and the initial entrance angle is $\theta_i = 15$.

Video S4. Simulation video showing the interaction of dumbbells with a wall with asymmetric stiffnesses of $k_L = 90$ and $k_R = 9$ in a 40 time units interval. The corresponding trajectories of dumbbells are plotted in Fig. 7b.
